# The endophytic bacteria isolated from elephant grass (*Pennisetum purpureum* Schumach) promote plant growth and enhance salt tolerance of Hybrid Pennisetum

**DOI:** 10.1186/s13068-016-0592-0

**Published:** 2016-09-02

**Authors:** Xia Li, Xiaoyan Geng, Rongrong Xie, Lei Fu, Jianxiong Jiang, Lu Gao, Jianzhong Sun

**Affiliations:** 1 Biofuels Institute, School of the Environment and Safety Engineering, Jiangsu University, 301 Xuefu Road, Zhenjiang, 212013 Jiangsu China; 2 BGI Zhenjiang Detection Co., LTD, 345 Gangnan Road, Zhenjiang, 212028 Jiangsu China

**Keywords:** Endophytic bacteria, Elephant grass, Plant growth promoting properties, Plant growth promoting effect, Salt stress tolerance, Hybrid Pennisetum

## Abstract

**Background:**

Elephant grass (*Pennisetum purpureum* Schumach) and Hybrid Pennisetum (*Pennisetum americanum* × *P. purpureum* Schumach) are tall, fast-growing perennial C4 bunchgrasses that have been in recent developed as the most appropriate biomass feedstock in many countries for exploring various biofuel products. However, the challenges of increasing plant biomass yield and enhancing their stress tolerance, especially on marginal lands, have been existed for a long while. In the past several years, bacterial endophytes used as bio-fertilizers for improving crop production have offered an opportunity to facilitate high biomass yield of energy crops in a more sustainable manner.

**Results:**

A total of 16 endophytic bacteria strains were isolated and purified from the roots of elephant grass, which were classified into four bacterial genera: *Sphingomonas, Pantoea, Bacillus,* and *Enterobacter*. Four strains, pp01, pp02, pp04, and pp06, represented four different genera, were then selected and tested in vitro for their plant growth promoting properties, effects on plant growth and salt stress tolerance of Hybrid Pennisetum. The inoculation with these four bacterial mixture demonstrated a significant plant growth promotion for Hybrid Pennisetum from the normal to salt stress conditions at 0, 50, 100, and 200 mM NaCl, respectively. The highest promotion rate for biomass yield was 116.01 and 81.72 % for shoot fresh weight and dry weight, respectively. The bacterial strains tested were shown to solubilize insoluble phosphate, fix nitrogen, produce indole acetic acid and ammonia, but only strains from *Sphingomonas*, *Bacillus*, and *Enterobacte*r can produce siderophore. In addition, the endophyte strains tested were all able to successfully colonize the roots of Hybrid Pennisetum, reaching upto 12.12 ± 0.98 CFU/g fresh roots at the 3rd day of inoculation.

**Conclusion:**

The four endophytic bacteria from elephant grass significantly promoted plant growth and biomass yield, alleviated the harmful effects of salt stress on Hybrid Pennisetum. These bacteria have indicated some unique properties that are very valuable for exploiting bio-inoculants aiding in the efforts to establish a sustainable and large-scale feedstock production system for Hybrid Pennisetum, particularly, on the saline marginal lands.

**Electronic supplementary material:**

The online version of this article (doi:10.1186/s13068-016-0592-0) contains supplementary material, which is available to authorized users.

## Background

Elephant grass (*Pennisetum purpureum* Schumach) is a tall, fast-growing perennial C4 bunchgrass discovered in 1905 in tropical Africa as a forage crop. Due to its high biomass productivity, 40~80 tons of dry biomass per hectare per annum, elephant grass was first studied as a biomass source for energy in the mid-90s [1–3]. In addition, it requires very little supplementary nutrients for growth and can be harvested up to four times a year, which makes this plant one of the most prospective crops for energy use [4]. In Colombia, elephant grass is presented as the most appropriate one for exploration in biofuel production with green forage yields between 360 and 400 ton per hectare per year obtained ethanol yields of 466.9 L/dry ton [5]. Hybrid Pennisetum is a highly sterile inter-specific Hybrid Hybridized from *Pennisetum americanum* (L.) Leeke and *Pennisetum purpureum* Schumach, which showed improvement in yield and forage quality over the parent species [6, 7]. With the advantages of high yield, high resistance to adverse conditions, quick regeneration capacity, free from pests and diseases, it has been widely cultivated in tropical and subtropical areas mainly used as forage crop [7–9]. Currently, Hybrid Pennisetum has been increasingly used as a promising energy crop for the production of ethanol, electric power and biogas [10–14].

It has been well known that one of the critical socioeconomic issues with the increasing use of biofuels is the competition of agricultural resources between energy crops and food crops [15]. One way to solve the conflict is to grow biofuel feedstocks on marginal lands that are not suitably applied for food crops [16]. But, the low biomass yield and a relatively high product cost have restricted a large-scale cultivation of the elephant grass and Hybrid Pennisetum on marginal lands. Thus, it is necessary to develop a novel technology to enhance the biomass yield in a cost effective and sustainable way adapted to various marginal lands.

In recentl years, bacterial endophytes used as bio-fertilizers for improving crop production are gaining strong status among agronomists and environmentalists because they would significantly reduce chemical input into the environments [17, 18]. Thus, the exploitation of plant growth promoting endophytes (PGPEs) as one of the best options to increase biomass yield of the energy crops on marginal lands has become a hot research subject with more attention both from academia and industry [16]. For instance, *Bacillus* sp. SLS18 promoted the biomass production of sweet sorghum [18]. The growth of poplar tree was improved up to 60 % after inoculation with different endophytic strains [15]. Both mycorrhizal fungi and bacterial endophyte have been reported enhancing biomass production in switchgrass [19, 20]. Endophytic bacteria are the unique microorganisms that colonize the internal tissues of plants latently or actively without substantively harming hosts [21–23]. Certain bacterial endophytes can promote plant growth and health under normal or adverse conditions, which were considered to be PGPEs. The PGPEs promote plant growth by various mechanisms include production of phytohormones [24–26], siderophores [27, 28], 1-aminocyclopropane-1-carboxylic acid (ACC) deaminase [29], nitrogen fixation [30, 31], and phosphates solution [24, 32]. So far, considerable number of PGPEs have been successfully isolated from a large diversity of plants and found to be beneficial for plant growth, yield and crop quality, including strains in the bacterial genera of *Acinetobacter, Alcaligenes*, *Arthrobacter, Azospirillium, Azotobacter, Azomonas, Bacillus, Beijerinckia, Burkholderia, Enterobacter, Erwinia, Flavobacterium, Klebsiella, Pseudomonas, Rhizobium and Serratia* [33–36]. Due to their beneficial effects on growth and health for host plants, PGPEs have the potential for use in the friendly, sustainable and organic agriculture [37, 38]. Hence, diverse endophytic bacteria are now being used worldwide as bio-inoculants to promote plant growth and development under normal and various stresses like heavy metals, herbicides, insecticides, fungicides, salinity, and so forth [17].

The objective of this study was to isolate endophytic bacteria from elephant grass, test them in vitro for their plant growth promoting properties, and evaluate their effects on plant growth and salt stress tolerance of Hybrid Pennisetum. This investigation would potentially offer an opportunity to exploit some valuable endophytic bacteria as biological inoculants to increasing the biomass yield of Hybrid Pennisetum, especially on salty marginal lands.

## Results

### Isolation and molecular identification of endophytic bacteria from elephant grass

A total of 16 endophytic bacteria, denoted as pp01~pp16, were isolated and purified from the roots of elephant grass. The 16S ribosomal RNA gene BLAST data suggested that they could be classified into four bacterial genera: *Sphingomonas, Bacillus, Pantoea,* and *Enterobacter*, where four representative isolates, pp01, pp02, pp04, and pp06, for each of the identified bacterial genera, were further described for their phylogeny as followings. For the pp01 strain, 16S ribosomal RNA gene amplicon showed its homology to partial sequence of *Sphingomonas paucimobilis* DSM 30198 (NCBI accessing number NR_104893.1) with 98 % identity. The pp02 isolate was close to *Bacillus megaterium* DSM319 (NCBI accessing number NC_014103.1) with 99 % homology. The pp04 isolate was identified as *Pantoea* sp. showed 99 % homology with the *Pantoea* sp. SAP16-1 (NCBI accessing number JN872526). The pp06 isolate 16S ribosomal RNA gene sequence showed homology to *Enterobacter ludwigii* strain KPS 4-2 (NCBI accessing number JQ308602) with 99 % identity. The 16S ribosomal RNA gene sequences of the four endophytic bacteria have been submitted to GenBank (NCBI) under the accession numbers KM220524, KM886123, KP271021 and KP271022.

### Effects of the selected bacterial inoculants on plant growth

**Fig. 1:**
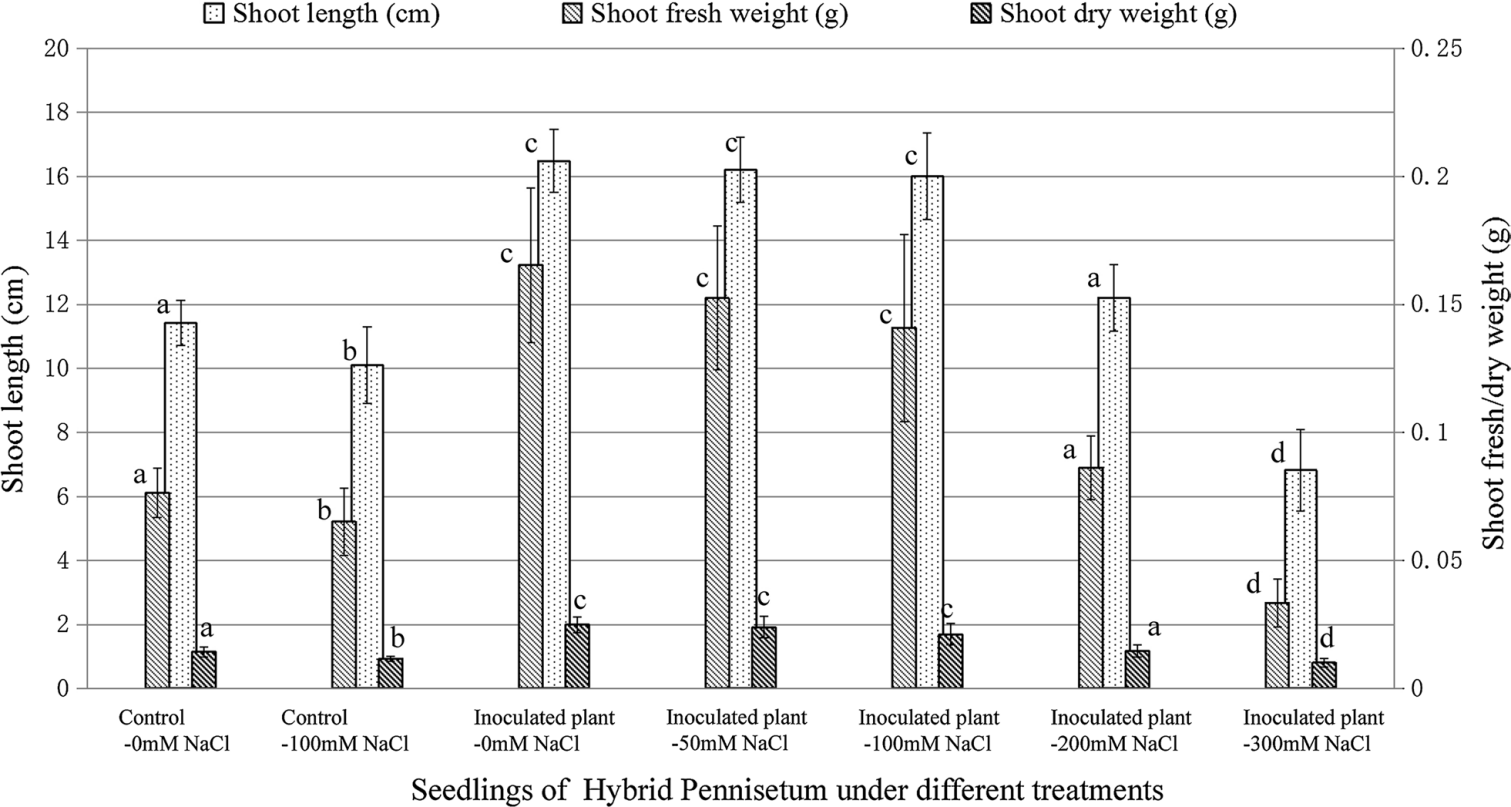
Effects of co-inoculation with four endophytic bacteria on growth parameters of Hybrid Pennisetum in vermiculite. The seedlings were co-inoculated with four endophytic bacteria, pp01, pp02, pp04, pp06, which grew in a plastic pot for 3 weeks. Dry weight was determined after samples were dried in oven at 80 °C for 24 h. (*Bars* with the* same letter* for each compared parameter did not differ significantly at *α* = 0.05, Duncan, *n* = 10–20. *Error bars* indicate ±1 SEM)

To evaluate the ability of the isolated endophytic bacteria to promote plant growth under a normal or a salt stress condition, the seedlings of Hybrid Pennisetum were inoculated with a mixture of the selected four bacterial strains, pp01, pp02, pp04, pp06. The results have showed that endophytic bacteria significantly promoted plant growth and its biomass yield under both normal and saline conditions, compared with those non-infected control groups (Fig. 1). Under normal condition, endophytic infection led to statistically significant increase in shoot length, shoot fresh weight and shoot dry weight by 44.38, 116.20, 74.19 %, respectively, when compared to those non-inoculated control plants (0 mM NaCl). When seedlings were exposed to a moderate salt stress, 100 mM NaCl, they showed an increased leaf chlorosis and a reduced plant growth response, leading to a decrease in shoots length, fresh and dry weight, at 11.48, 14.76 and 18.90 %, respectively, when compared to those under normal condition (0 mM NaCl). However, the inoculation of the four bacterial strains mixture (OD_600_ = 1, ~10^6^ to ~10^8^ CFU/mL, Additional file 1: Table S1) almost eliminated the observable detrimental effect at low (50 mM NaCl) and moderate (100 mM NaCl) salt stress, where the plants had the same growth promotion effects as those infected plants under normal stations, 0 mM NaCl. Our observations also indicated that at high salt stress, 200 mM NaCl, in spite of an observed reduction in promotion effects, the inoculated plants even grew better than those control groups at the normal condition, 0 mM NaCl. However, at a high salt stress, 300 mM NaCl, the effect was apparently decreased with the fact that seedling biomass yield was remained at a low level in spite of the existence of endophytes.

### The optimal concentration of endophytic inoculants to promote plant growth

**Fig. 2:**
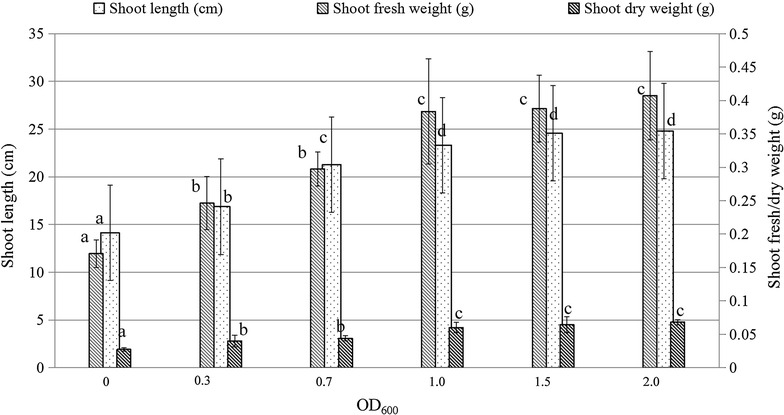
Effects of co-inoculation with four endophytic bacteria, pp01, pp02, pp04, pp06, at different concentrations on growth parameters of Hybrid Pennisetum in vermiculite. The seedlings were co-inoculated with four endophytic bacteria, which grew in a plastic pot for 3 weeks. Dry weight was determined after samples were dried in oven at 80 °C for 24 h. (*Bars* with the* same letter* for each compared parameter did not differ significantly at *α* = 0.05, Duncan, *n* = 10–20. *Error bars* indicate ±1 SEM)

The optimal concentration of the selected endophytic bacteria for inoculation was determined by inoculating Hybrid Pennisetum seedlings at different concentrations of endophytic inoculants that mixed with four different endophyte isolates, pp001, pp002, p004, p006 (OD_600_ at 0.3~2.0, ~10^5^ to ~10^8^ CFU/mL, Additional file 1: Table S1). The plant promoting effects, indicated by shoot length, shoot fresh and dry weight, were enhanced at a higher concentration of the endophytes, but no significant changes were observed when it is higher than 1.0 OD_600_ (Fig. 2). Apparently, the optimal threshold concentration was higher than 1.00 OD_600_. The potential highest promotion effects, represented by shoots length, shoot fresh weight, shoot dry weight, were recorded at 64.86, 124.69, 119.08 %, respectively, when compared to those non-infected control plants.

### Plant growth promoting (PGP) properties of endophytic bacteria

The four endophytic bacteria were assayed for their PGP properties that may play the important roles on plant growth and salt stress tolerance (Table 1). The results showed that all strains tested had at least two important PGP traits to be identified out of a serial of evaluations. Each strain tested had the ability to produce IAA at a range of 10.50–759.19 mg/L, where *Enterobacter* sp. demonstrated the highest value. The four strains tested all exhibited relatively high levels of ACC deaminase activity, where *Pantoea* sp. presented the highest ACC deaminase activity at 1106.66 ± 78.59 nmol a-ketobutyrate (KB)/h/mg. It is noteworthy that all strains tested can successfully grow in the Ashby nitrogen-free culture medium (without agar) as well as the Jensen medium, but exhibiting different levels of nitrogen fixing activities. Based on the degree of turbidity of Ashby medium, *Sphingomonas* sp. recorded the highest nitrogen fixing ability followed by *Pantoea* sp. among four tested endophytic bacteria. In addition, it is virtually consistent that all the four strains were positive for producing ammonia, which can be used by the plants as a source of nitrogen for their growth [39]. Besides, all the strains tested were also able to solubilize inorganic phosphate [Ca_3_(PO_4_)_2_] into a soluble form that is easily accessible by plants. Based on our further evaluation, those endophytic bacteria belonging to *Sphingomonas*, *Bacillus*, *Enterobacter* exhibited siderophore production ability, where *Bacillus* recorded the optimum siderophore 64.06 % units. Obviously, a mixed inoculant with four endophytic bacteria would simultaneously possess all the tested PGP properties that may maximize the effects of endophytes on plant growth promotion.

**Table 1:** PGP properties of the tested four endophytic bacterial strains

Strains	IAA production capacity	ACC deaminase activity (nmol α-KB/h/mg)	Nitrogen fixing capacity ^a^	Ammonia production capacity	Siderophore production capacity ([(Ar − As)/Ar] × 100 %)^b^	Inorganic phosphate solubilizing capacity
*Sphingomonas* sp. pp01	19.85 ± 1.67	524.82 ± 32.60	++++	+	10.33 ± 2.21	+
*Bacillus* sp. pp02	10.50 ± 2.19	225.20 ± 82.20	++	+	64.06 ± 7.27	+
*Pantoea* sp. pp04	40.88 ± 0.80	1106.66 ± 78.59	+++	+	−25.61 ± 2.50	+
*Enterobacterc* sp. pp06	759.19 ± 54.42	902.14 ± 34.99	+	+	21.11 ± 2.54	+

± standard error (SE); + positive; – negative

^a^Nitrogen-fixing capacity: + little; ++ low; +++ moderate; ++++ high

^b^ % siderophore units

### The root colonization ability of selected endophytic bacteria

**Fig. 3:**
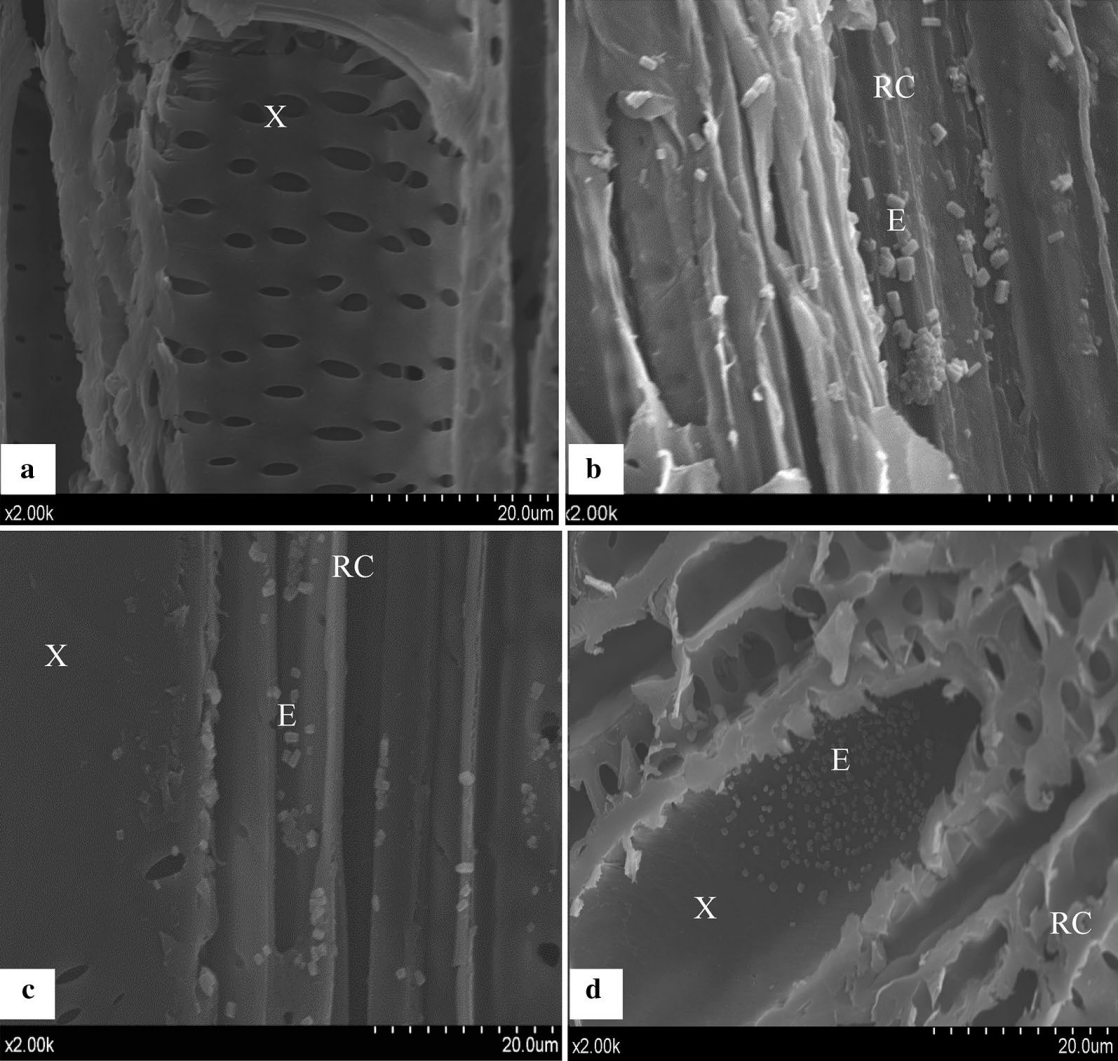
Locations of the endophytic bacteria resided in the tissue of the roots of inoculated Hybrid Pennisetum, which were imaged by SEM. **a** Control, no endophytic bacteria were detected in the tissues of non-inoculated host plants; **b** Treated plants, 3 days after inoculation, cells of endophytic bacteria were detected in the root cortex; **c** Treated plants, 7 days after inoculation; cells of endophytic bacteria were detected mostly in the root cortex and a few in the xylem vessel; **d** Inoculated plants, 14 days after inoculation, a large number of bacterial cells were already found in the xylem vessel. *RC* root cortex; *X* xylem vessel; *E* cell of endophytic bacteria

The interior colonization ability of those inoculated endophytes was analyzed by determining the titer of re-isolated endophytes using tissue homogenates from the inoculated plant roots sampled at different times. Each of the four selected endophytic bacteria strains could successfully colonize in its host plant tissues three days after inoculation, with the bacterial densities from 4.91 ± 0.43 to 12.12 ± 0.98 CFU/g fresh plant roots (Table 2). Following that inoculation processing, the bacterial population increased rapidly, where, on the 10th day of inoculation, *Pantoea* sp. pp04 demonstrated the highest density of 158.16 ± 11.77 CFU/g fresh plant roots. This observation indicated that the endophytic bacteria were able to colonize the host plant roots on the 3rd day after inoculation, and then reproduced rapidly in its population to achieve an extensive colonization. This was verified by performing scanning electron microscopy (SEM) inspection of the roots for those inoculated Hybrid Pennisetum sampled at 3, 7, and 14 days after inoculation (Fig. 3). On the 3rd day after inoculation, cells of endophytic bacteria were successfully detected in the root cortex. On the 7th day after inoculation, bacteria cells were detected mostly in the root cortex and a few in the xylem vessel. On the 14 day after inoculation, a large number of bacterial cells were already found in the xylem vessel. Clearly, it has been verified by SEM inspection that endophytic bacteria were already colonized in the central parts of the plant roots 7 days after inoculation.

**Table 2:** Number of endophytic bacterial colonies isolated from the roots of plants after inoculation

Endophytic bacteria strain	3 days after inoculation	10 days after inoculation
*Sphingomonas* sp. pp01	11.7 ± 1.76	28.9 ± 5.84
*Bacillus* sp. pp02	4.91 ± 0.43	21.31 ± 1.59
*Pantoea* sp. pp04	12.12 ± 0.98	158.16 ± 11.77
*Enterobacter* sp. pp06	5.61 ± 0.604	42.43 ± 4.62

Mean and standard error of three replicas per treatment, values in CFU/g plant roots

## Discussion

It has been confirmed in recent years that PGPEs have the ability to colonize host plant’s interior tissues and further build a beneficial symbiotic association with their host plants to improve host plant growth and stress tolerance [40, 41]. This function would indeed facilitate a higher biomass production of energy crops, in a more sustainable manner, especially on infertile and marginal lands [17]. In this study, we isolated a total of 16 strains of endophytic bacteria from elephant grass, which were sorted into four bacteria genera, *Sphingomonas*, *Bacillus*, *Pantoea*, and *Enterobacter* by a phylogenic analysis. Four representative strains, pp01, pp02, pp04, pp06, were selected and systematically investigated for their PGP properties, ability of root colonizing, effects of plant growth promoting on Hybrid Pennisetum. With respect to PGP properties, the four endophytic bacteria possessed a serial of relevant properties, including IAA production, siderophore production, nitrogen fixation, ammonia production, inorganic phosphate solubilization, and ACC deaminase activity (Table 1). It has also been confirmed that the endophytic bacteria tested can successfully colonized in vivo the host plants (Table 2) and stimulate a significant increase in shoot length, shoot fresh weight and shoot dry weight in Hybrid Pennisetum compared to the un-inoculated controls under both normal and saline conditions (upto 200 mM NaCl) (Fig. 1). Obviously, these results suggest that the endophytic bacteria we have isolated can be of great value in enabling Hybrid Pennisetum to grow better with a higher biomass production on arable lands or even those infertile and saline marginal lands.

PGPEs can stimulate plant growth directly or indirectly with different and unique mechanisms, such as with production of plant hormones, enhancement of nutrient uptake and stress tolerance, bio-control of plant pathogen [34, 42, 43]. A particular endophytic bacterium may promote plant growth and development using one or more of these mechanisms at various times during the life cycle of the plant [44]. In this research, each of the endophytic bacteria tested possessed at least two or more properties that were linked to the plant growth promoting activities, including the ability of IAA production, siderophore production, nitrogen fixation, ammonia production, inorganic phosphate solubilization, or ACC deaminase activity. Therefore, the inoculants combined with different endophytic bacteria may possibly possess various PGP properties that can be complementary with each other to offer them an enhanced power to optimally facilitate the growth of plant by utilization of various mechanisms at different times during the life cycle of host plants. The tested endophytic bacteria demonstrated ability to produce IAA and siderophores, solubilize inorganic phosphate, which could facilitate the growth of plant and salt resistance, in agreement with early investigations being reported [45–50]. ACC deaminase activity is a common characteristic of PGPEs, which promotes plant growth and eases plant stress by reducing the ethylene level via degrading ACC (the precursor of ethylene) to ammonia and ∝-ketobutyrate [51–54]. Apparently, the ACC deaminase activity of the endophytes tested has been shown to be virtually high, with 225.2–1106.66 nmol ∝-KB/h/mg. It is reported that a low level of ACC deaminase activity, approximately higher than 20 nmol ∝-KB/h/mg is sufficient for a bacterium to promote plant growth as a PGPE [44]. Moreover, the plants inoculated by ACC deaminase containing PGPE are found more resistant to high salt stress [55]. Hence, it is proposed that the high ACC deaminase activity of the endophytes tested is a main contributed property to enhance the plant growth and salt tolerance of Hybrid Pennisetum seedlings in vitro.

However, it was also suggested in early reports that mycorrhizal fungi would also alleviate salt stress by absorption of mineral nutrients (mainly N, P, K) [56] and osmotic regulation in the inoculated plants [57]. Inoculation of plants by Arbuscular mycorrhizal fungi exhibited a lower concentration of Na^+^ and a higher concentration of N, P, K, Mg^2+^ than non-inoculated plants [58–61]. In saline soil, chlorophyll contents, proline content, total soluble protein content, acid and alkaline phosphatase activities were also observed higher in arbuscular mycorrhizal fungus inoculated plants, which could be important for salt alleviation in plant when growing in saline soils [62–65]. At present, the mechanisms governing the absorption of mineral nutrients and ionic balance in inoculated seedlings are still unknown. Much work is further required to reveal the mechanisms that regulate the beneficial effects of the endophytes isolated from elephant grass.

It is well known that salinity stress will restrict plant growth through the low uptake of water and nutrients due to the ion-toxic effects of Na^+^ [66–68]. In recent years, endophytes have been used as one of the practical measurements to alleviate salt stress and improve plant health and yield in saline soil [40, 69, 70]. *Pseudomonas fluorescens* isolate, TDK1, showed great performance in improving the plant growth for groundnut seedlings under salt stress in vitro [41]. Mycorrhizal inoculation significantly increased growth response of wheat plants in saline soil [71]. In the present work, the four endophytes isolated from elephant grass were combined together to evaluate their plant growth promoting effects under normal or saline conditions. From this observation, when endophytes were inoculated to plant seedlings growing under normal conditions, the plant growth and biomass yield of Hybrid Pennisetum were significantly enhanced by 44.38, 116.20, and 74.19 % in plant shoot length, shoot fresh weight and shoot dry weight, respectively. Interestingly, the detrimental effect of low (50 mM NaCl), moderate (100 mM NaCl) salt stress, and (200 mM NaCl) high salt stress was significantly alleviated by the combined endophytic inoculants, where the shoot length, shoot fresh weight and shoot dry weight were dramatically enhanced up to 58.30, 116.01 and 81.72 %, respectively, compared to the un-inoculated control plants under moderate (100 mM NaCl) salt stress. Therefore, it has been suggested that these endophytic bacteria were very effective, and actually served as the bio-inoculants to improve plant growth and biomass yield of Hybrid Pennisetum when growing on infertile and saline lands. But, it is noteworthy that the plant promotion effects mentioned here were indeed recorded from a co-inoculated treatment combined with four different bacterial strains, and it is remained to be straighten out the plant promoting effects for each individual bacterial strain. Additionally, it has also been reported that the plant promoting microorganisms showing a decreased performance from the laboratory to the field, which may be attributed to a number of factors including competition for nutrients with other soil microbiota [20, 72]. Thus, much work is still required to evaluate the PGP effects in the field conditions to get a reliable and stable performance.

Bacterial infection and the subsequent colonization in host plant tissues are critical for the eventual beneficial impact of endophytes on plant growth [42]. The colonizing ability may confer endophytes more favorable environment under various biotic and abiotic stresses and a competitive advantage to native soil bacteria compared to rhizospheric bacteria [73]. Thus, it has been suggested that to be an excellent plant growth promoting inoculants, endophytic bacteria must be capable of colonizing in the interior tissues of a host plant [74]. The endophytic bacteria reported here suggested an active invasion and proliferation advantage in the roots of 1-week-old seedlings. Three days after colonization, we could clearly visualize bacterial population in the cortex parts of the plant roots after a SEM inspection, where the bacterial densities were recorded from 4.91 ± 0.43 to 12.12 ± 0.98 CFU/g fresh plant roots. Thus, the endophytic bacteria had the potential to survive within the tissues of Hybrid Pennisetum, suggesting that they can be the suitable candidates served as the endophytic bio-inoculants. However, the population densities were still lower than our expectation, which may be attributed to the mature root material being sampled for recovery assay. This result agreed with the previous findings that had indicated that low colonization was detected in the mature parts of the rice roots inoculated by *Pantoea agglomerans* YS19 [75]. It is also reported that the root colonization ability for endophytic bacteria would exhibit a lower level on a non-sterile soil condition than that in a gnotobiotic environment for establishing themselves on plant roots, which could be attributed to a competition for nutrients with other soil microbiota [76]. Therefore, it is further required for us to cope with the challenges under a field condition to enhance colonization capability of endophytes that can be potentially applied to various soil environments.

## Conclusion

In conclusion, our findings reported that the co-inoculation of four selected endophytic bacterial strains that were successfully isolated from elephant grass significantly alleviated the harmful effects of salt stress, promoted plant growth and biomass yield on Hybrid Pennisetum in vitro. Each of the bacterial strains tested showed at least two or more PGP properties, including the ability of IAA production, siderophore production, nitrogen fixation, ammonia production inorganic phosphate solubilization, or ACC deaminase activity. Thus, these evaluations suggest a potential utilization of these beneficial bacterial endophytes as promising candidates for bio-inoculants, which may aid in building a sustainable feedstock production system for Hybrid Pennisetum, especially on those infertile or marginal saline lands. However, further investigations are also required to reveal the relevant mechanisms on infection and colonization, plant growth promoting effects, as well as their unique salt stress tolerance property, which will further optimize the beneficial effects of the endophytic bacteria on their host plants in a field application.

## Methods

### Plant material and isolation of bacterial endophytes

The elephant grass elite Sumu No. 2 was grown in the nursery bed at the Jiangsu University in Jiangsu Province, Eastern of China. Endophytic bacteria were isolated from the healthy and asymptomatic roots of elephant grass based on the method described by the Sturz et al. [77] and Surette et al. [78] with a minor modification. Briefly, roots were washed thoroughly under tap water for 10 min to remove any adhering soil, dipped in 10 % of commercial bleach (5.25 % available chlorine) for 3 min, then transferred to a 3 % hydrogen peroxide solution for 3 min, and finally rinsed three times with sterile water. A 0.002 % solution of Tween 20 was added to the first rinse solution. To ascertain that the surface disinfection process was successful, an aliquot of 100 μL final wash was inoculated in LB medium for sterility check. Then, root tissues were macerated using a mortar and pestle in a small volume of sterile phosphate buffered saline (PBS, pH 7.4). This suspension was plated on LB medium and incubated at 28 °C for 48–72 h.

### Bacterial identification using 16S rRNA sequences

The bacterial strains were characterized by 16S rRNA gene (rDNA) sequencing analysis. PCR were performed from overnight grown cells using universal primers (27F: AGAGTTTGATCCTGGCTCA and 534R: ATTACCGCGGCTGCTGG) [79]. The amplification was performed in a thermocycler programmed as follows: 95 °C for 3 min; 34 cycles of 95 °C for 30 s, 55 °C for 30 s, 72 °C for 1 min; 72 °C for 10 min; 4 °C for storage. The PCR amplicon was purified with TaKaRa Agarose Gel DNA Purification Kit (TaKaRa, China) and sequenced (Sangon Inc., China). Partial 16S rDNA sequences obtained were analyzed using the BLAST tool in the NCBI website.

### Inoculation of Hybrid Pennisetum for assessment of colonization and plant growth

A pot experiment was set up in growth chamber to assess the growth promotion effects and their colonization ability on Hybrid Pennisetum by inoculation of the selected four bacterial endophytes, pp01, pp02, pp04, pp06, in combination. For the homogeneity of host plants, seeds of Hybrid Pennisetum were used for analyzing the efficacy of selected bacterial endophytes to promote plant growth and salt tolerance, and their colonizing ability for host plants. They were surface sterilized with the following protocol: 70 % alcohol 5 min; washed with sterile water; 10 % H_2_O_2_ 20 min; washed with 50 mL half-strength Hoagland’s nutrient solution. The sterilized seeds were then placed on the agar plate for germination at 25 °C. After 2 days, seedlings were sown into plastic pot filled with 40 g sterilized vermiculite moistened with sterilized water. The seedlings were then grown in the growth chamber at 25 °C with a 16 h light/8 h dark photoperiod. For inoculation of the seedlings, bacterial cultures were grown in 100 mL LB medium at 30 °C on a rotary shaker (100 rpm) till they reached the proper concentration (OD_600_ value of 0.3~2.0, ~10^5^ to ~10^8^, Additional file 1: Table S1). Bacterial cultures were then harvested by centrifugation (6000 rpm, 10 min), washed twice with sterile water and re-suspended in sterile water. The roots of 1-week-old seedlings were incubated with the selected four bacterial endophytes that poured off 50 mL resulting suspensions (pp01:pp02:pp04:pp06 = 1:1:1:1) into the vermiculite of the pot. Three days later, root samples were checked for endophyte infestation according to the same method used for the endophytic bacteria isolation. The recovered bacteria colonies were verified by performing 16S rDNA PCR amplification. Then, the 50 mL NaCl solution at one of the concentrations, 50, 100, 200 and 300 mM, was added to the pot every 4 days to expose seedlings to different salt stress. We added quite a lot of NaCl liquid over the course of the experiment to keep the vermiculite moist. The excess liquid was kept in the outer layer of the pot to ensure that the saline concentration is maintained at a constant level until the seedlings were watered next time. The control seedlings were watered only with the same volume of sterile water. After 3–4 weeks, whole plants were carefully removed from the pots, and the soil was removed from the roots. Growth parameters such as height, fresh weight and dry weight (oven dried at 80 °C for 24 h) of the plants were measured. Each experiment was replicated at least three times and each treatment had 10–20 biological replicates.

### The colonization ability

Seedlings of Hybrid Pennisetum were inoculated with the selected four endophytic bacteria based on the same method mentioned above. To study the interior colonization ability of the four endophytic bacteria strains, SEM and the inoculum recovery were performed. On the 3rd and 10th day, after the seedlings were incubated by the four bacterial strains, respectively, plant roots were collected, surface-sterilized, macerated and plated on LB medium in terms of the same method used for isolating the endophytic bacteria. The bacterial colonies were counted after incubation for 3 days at 30 °C. Normalization of counts was carried out based on the root weight. These recovered inoculants were then verified by performing 16S rDNA PCR amplification. Triplicates were performed for each sampling time, and all values were averaged for a means of these measurements. Control plants were also set up in parallel at each sampling time.

For examination with SEM, the fresh root samples from both control plants and treated plants that are inoculated with the combined four endophytic bacteria were excised at 3, 7, 14 days after inoculation. The plant root samples were then processed as described previously with a minor modification [80]. First, the samples were rinsed with sterilized double-distilled water for three times and then fixed with 2.5 % (v/v) glutaraldehyde for 8 h at 4 °C. The fixed roots were washed with 0.1 M phosphate buffer 3 times for 15 min each. Afterwards, the roots were dehydrated in a gradient ethanol series, 50, 70, 80, 90 and 100 %, each for 15 min, followed by immersing the roots in 50 and 100 % isopentyl acetate for 1 and 4 h, respectively. Subsequently, samples were dried in Virtis Freeze Dryer (−55 °C; Gardiner, NY, USA) and coated with gold palladium in a sputter-coater (Balzers SCD 040, Liechtenstein). Finally, the samples were observed under a field emission SEM (JEOL JSM7001F, Tokyo, Japan).

### Evaluation of some important PGP properties

#### Nitrogen fixing capacity

The nitrogen fixing capacity of the four endophytic bacteria were determined by their growth in the nitrogen-free Ashby medium and Jensen medium [81, 82]. Bacteria were inoculated in 5 mL Ashby medium (without agar) in 45 mL test tube on a rotary shaker (125 rpm) at 30 °C for about 7 days to observe if the culture became turbid. The colonies of the four endophytic bacteria were inoculated on the Jensen medium, respectively, at 30 °C for 4 days to observe the presence of growth. The non-inoculated media served as control.

#### Ammonia (NH_3_) production capacity

The selected endophytic bacteria were evaluated for their production capacities of ammonia. Four different bacterial isolates were inoculated into 10 mL peptone water (Peptic digest 10 g/L, NaCl 5 g/L, dH_2_O, 1000 mL, pH 7.2), respectively, for 48–72 h at 30 °C. Then 0.5 mL of Nessler’s reagent (K_2_HgI_4_ and NaOH or KOH) was then added to the culture. The color change from brown to yellow was considered as a positive result. Non-inoculated medium was used as the control [83].

#### ACC deaminase capacity

ACC deaminase capacity was assayed with the method from Saravanakumar and Samiyappan [41], which measures the amount of ∝-ketobutyrate being produced when the enzyme ACC deaminase cleaves ACC by comparing the absorbance at 540 nm of a sample to a standard curve of ∝-ketobutyrate ranging between 0.1 and 1.0 nmol [84].

#### Indole acetic acid (IAA) production

IAA production was measured according to Sheng et al. [85]. The selected bacteria were cultured in sucrose-minimal salts (SMS) medium supplemented with 0.5 mg/mL of tryptophan for 4 days. One milliliter of bacterial culture was mixed with 2 mL of Salkowski’s reagent [86] and then incubated at room temperature for 20 min until a pink color developed in the suspension. Then, the absorbance was measured at 535 nm. The IAA concentration was determined from IAA standard curve following the linear regression analysis.

#### Siderophore production

Siderophore production ability was investigated based on the chrome azurol-S analytical method [87]. Bacterial cultures were grown in King B medium without phosphate at 30 °C on a rotary shaker (200 rmp) for 5 days and the cell free culture supernatant was harvested by centrifugation (10,000 rpm) for 10 min. One milli litre of cell free culture supernatant was mixed with 1.0 mL of CAS assay solution and incubated in a dark environment at room temperature for 1 h. The non-inoculated supernatant was used as reference. Then, the absorbance was measured at 630 nm. The samples, denoted as “As”, are supposed to have a lower absorbance at 630 nm than that of the reference, denoted as “Ar”. The decrease in absorbance at 630 nm was recorded, and the values were then compared with the OD630 read from reference (Ar). Siderophore units were defined as: % siderophore units = (Ar − As)/Ar] × 100 % [88].

#### Phosphate-solubilizing capacity

The phosphate solubilizing capacities were analyzed in the National Botanical Research Institute’s phosphate (NBRIP) medium [89]. 1.5 % of tricalcium phosphate [Ca_3_(PO_4_)_2_] was used as the inorganic phosphate source. Then, the plates were incubated at 28 °C for 72 h, and the formation of clear halo around the colonies was an indication of inorganic phosphate solubilization.

### Statistical analysis

The PGP effects of endophytic bacteria on growth parameters of Hybrid Pennisetum at different salt stress and different inoculating concentrations were analyzed by one way ANOVA using SPSS version 17.0, and the means were separated by Duncan Multiple Range Test (DMRT) at the 0.05 level of significance.

## Additional file

10.1186/s13068-016-0592-0 The densities of the four endophytic bacterial strains corresponding to different OD600 values 0.3–2.0.

## Abbreviations

IAA: indole acetic acid
PGPE: plant growth promoting endophyte
ACC: 1-aminocyclopropane-1-carboxylic acid
NCBI: The National Center for Biotechnology Information
SEM: scanning electron microscopy
PBS: phosphate buffered saline
PCR: polymerase chain reaction
DNA: deoxyribonucleic acid
BLAST: the basic local alignment search tool
SMS: sucrose-minimal salts
CAS: chrome azurol-S
NBRIP: National Botanical Research Institute’s phosphate medium

## References

[1] Nagwani M. Calcium hydroxide pretreatment of biomass. M.S. thesis. Texas A & M University, 1992.

[2] Woodard KR, Prine GM (1993). Dry matter accumulation of elephant grass, energycane, and elephant millet in a subtropical climate. Crop Sci.

[3] Fontoura CF, Brandão LE, Gomes LL, Bastian-Pinto C. Modeling electricity prices in Brazil: application in an elephant grass biomass power plant with switch option. In 16th annual international conference on real options. London; 2012.

[4] Osava M. Capim Elefante, Novo Campeão em Biomassa no Brasil. AgrosoftBrasil, Pereira. http://www.agrosoft.org.br/?q=node/26484. Accessed July 20, 2013.

[5] Cardona EM, Rios LA, Peña JD (2012). Disponibilidad de variedades de pastos y forrajes como potenciales materials lignocelulósicos para la producción de bioetanol en Colombia. Inf Tecnol.

[6] Muldoon DK, Pearson CJ (1979). The Hybrid between *Pennisetum americanm* and *P purpureum*. Herbage Abs..

[7] Premaratne S, Premalal GG (2006). Hybrid Napier (*Pennisetum perpureum* × *Pennisetum americarnum*) var CO-3: a resourceful fodder grass for dairy development in Sri Lanka. J Agric Sci.

[8] Yu D, Wang N (2007). The propagation of *Pennisetum dydridum* in karst area and its all-round benefit. Guizhou Sci..

[9] Lowe AJ, Thorpe W, Teale A, Hanson J (2003). Characterisation of germplasm accessions of Napier grass (*Pennisetum purpureum* and *P purpureum* × *P. glaucum* Hybrids) and comparison with farm clones using RAPD. Genet Resour Crop Evol.

[10] Chang SH, Muzi SJ (2009). Giant king grass: the new biomass for green energy.

[11] Gao F, Liu B, Sun Q (2011). Dilute acid pretreatment and fermentation of energy grass. Southwest Chin J Agr Sci..

[12] Takara D, Khanal SK (2011). Green processing of tropical banagrass into biofuel and biobased products: an innovative biorefinery approach. Bioresource Technol..

[13] Wu JZ, Zhang JL, Pan YM, Liu ZW, Zhong XX (2014). Changes of the content and biomass of cell wall components and calculated ethanol yields in *Pennisetum purpureum* and Hybrid Pennisetum during the growing period. Acta prataculturae sinica..

[14] Huang QL, Huang XS, Chen ZD, Zhong ZM, Feng DQ (2012). Research on the biogas production of Pennisetum. Anhui Agri Sci Bull..

[15] Safiyh T, Craig G, Sébastien M (2009). Genome survey and characterization of endophytic bacteria exhibiting a beneficial effect on growth and development of poplar trees. Appl Environ Microbiol.

[16] Weyens N, van der Lelie D, Taghavi S, Newman L, Vangronsveld J (2009). Exploiting plant–microbe partnerships to improve biomass production and remediation. Trends Biotechnol.

[17] Ahemad M, Kibret M (2014). Mechanisms and applications of plant growth promoting rhizobacteria: current perspective. J King Saud Univ Sci..

[18] Luo SL, Xu TY, Chen L, Chen JL, Rao C, Xiao X, Wan Y, Zeng GM, Long F, Liu CB, Liu YT (2012). Endophyte-assisted promotion of biomass production and metal-uptake of energy crop sweet sorghum by plant-growth-promoting endophyte *Bacillus* sp. SLS18. Appl Microbiol Biotechnol.

[19] Ghimire SR, Charlton ND, Craven KD (2009). The mycorrhizal fungi, *Sebacina vermifera*, enhances seed germination and biomass production in switchgrass (*Panicum viratum* L.). BioEnergy Res..

[20] Kim S, Lowman S, Hou G, Nowak J, Flinn B, Mei C (2012). Growth promotion and colonization of switchgrass (*Panicum viratum*) cv. Alamo by endophyte *Burkholderia phytofirmans* strain PsJN. Biotechnol Biofuels.

[21] Hallmann J, Hallmann AQ, Mahaffee WF, Kloepper JW (1997). Bacterial endophytes in agricultural crops. Can J Microbiol.

[22] Misaghi IJ, Donndelinger CR (1990). Endophytic bacteria in symptom free cotton plants. Phytopathology..

[23] Azevedo JL, Maccheroni W, Pereira JO, Araujo WLD (2000). Endophytic microorganisms: a review on insect control and recent advances on tropical plants electron. J Biotech..

[24] Ahemad M, Khan MS (2012). Evaluation of plant growth promoting activities of rhizobacterium *Pseudomonas putida* under herbicide-stress. Ann Microbiol.

[25] Lee S, Flores-ncarnacion M, Contreras-Zentella M, Garcia-Flores L, Escamilla JE, Kennedy C (2004). Indole-3-acetic acid biosynthesis is deficient in Gluconacetobacter diazotrophicus strains with mutations in cytochrome C biogenesis genes. J Bacteriol.

[26] Zahir ZA, Arshad M, Frankenberger WT (2004). Plant growth promoting rhizobacteria application and perspectives in agriculture. Adv Agron.

[27] Jahanian A, Chaichi MR, Rezaei K, Rezayazdi K, Khavazi K (2012). The effect of plant growth promoting rhizobacteria (PGPR) on germination and primary growth of artichoke (*Cynara scolymus*). Int J Agric Crop Sci..

[28] Rajkumar M, Ae N, Prasad MNV, Freitas H (2010). Potential of siderophore-producing bacteria for improving heavy metal phytoextraction. Trends Biotechnol.

[29] Glick BR, Todorovic B, Czarny J, Cheng Z, Duan J, McConkey B (2007). Promotion of plant growth by bacterial ACC deaminase. Crit Rev Plant Sci.

[30] Triplett EW (1996). Diazotrophic endophytes: progress and prospects for nitrogen fixation in monocots [J]. Plant Soil.

[31] Dobereiner J (1997). Biological nitrogen fixation in the tropics: social and economic contributions. Soil Biol Biochem.

[32] Ma Y, Rajkumar M, Luo Y, Freitas H (2011). Inoculation of endophytic bacteria on host and non-host plants-effects on plant growth and Ni uptake. J Hazard Mater.

[33] Rodriguez H, Fraga R (1999). Phosphate solubilizing bacteria and their role in plant growth promotion. Biotechnol Adv.

[34] Sturz AV, Christie BR, Nowak J (2000). Bacterial endophytes: potential role in developing sustainable systems of crop production. Crit Rev Plant Sci.

[35] Sudhakar P, Chattopadhyay GN, Gangwar SK, Ghosh JK (2000). Effect of foliar application of Azotobacter, Azospirillum and Beijerinckia on leaf yield and quality of mulberry (*Morus alba*). J Agric Sci.

[36] Berg G (2009). Plant–microbe interactions promoting plant growth and health: perspectives for controlled use of microorganisms in agriculture. Appl Microbiol Biotechnol.

[37] Lucy M, Reed E, Glick BR (2004). Applications of free living plant growth-promoting rhizobacteria. Antonie Van Leeuwenhoek.

[38] O’Connell PF (1992). Sustainable agriculture—a valid alternative. Outlook Agric..

[39] Ahmad F, Ahmad I, Khan MS (2008). Screening of free-living rhizospheric bacteria for their multiple plant growth-promoting activities. Microbiol Res.

[40] Mayak S, Tirosh T, Glick BR (2004). Plant growth promoting bacteria that confer resistance in tomato and pepper to salt stress. Plant Physiol Biochem.

[41] Saravanakumar D, Samiyappan R (2007). ACC deaminase from *Pseudomonas fluorescens* mediated saline resistance in groundnut (*Arachis hypogea*) plants. J Appl Microbiol.

[42] Mei C, Lara-Chavez A, Lowman S, Flinn B, Luo H, Wu Y (2014). The use of endophytes and mycorrhizae in switchgrass biomass production. Compendium of bioenergy plants: switchgrass.

[43] Bibi F, Yasir M, Song GC, Lee SY, Chung YR (2012). Diversity and characterization of endophytic bacteria associated with tidal flat plants and their antagonistic effects on oomycetous plant pathogens. Plant Pathol J..

[44] Glick BR (2003). Phytoremediation: synergistic use of plants and bacteria to clean up the environment. Biotechnol Adv.

[45] Jha P, Kumar A (2009). Characterization of novel plant growth promoting endophytic bacterium Achromobacter xylosoxidans from wheat plant. Microb Ecol.

[46] Jha B, Gontia I, Hartmann A (2012). The roots of the halophyte Salicornia brachiata are a source of new halotolerant diazotrophic bacteria with plant growth-promoting potential. Plant Soil.

[47] Glick BR (1995). The enhancement of plant growth by free living bacteria. Can J Microbiol.

[48] Alexander DB, Zuberer DA (1991). Use of chrome azurol S reagents to evaluate siderophore production by rhizosphere bacteria. Biol Fertil Soils.

[49] Burd GI, Dixon DG, Glick BR (2000). Plant growth promoting bacteria that decrease heavy metal toxicity in plants. Can J Microbiol.

[50] Nabti E, Sahnoune M, Ghoul M, Fischer D, Hofmann A, Rothballer M, Schmid M, Hartmann A (2010). Restoration of growth of durum wheat (*Triticum durum* var. waha) under saline conditions due to inoculation with the rhizosphere bacterium *Azospirillum brasilense* NH and extracts of the marine alga *Ulva lactuca*. J Plant Growth Regul.

[51] Glick BR, Penrose DM, Li J (1998). A model for the lowering of plant ethylene concentrations by plant growth-promoting bacteria. J Theor Biol.

[52] Penrose DM, Moffatt BA, Glick BR (2001). Determination of 1-aminocyclopropane-1-carboxylic acid (ACC) to assess the effects of ACC deaminase-containing bacteria on roots of canola seedlings. Can J Microbiol.

[53] Glick BR (2004). Bacterial ACC deaminase and the alleviation of plant stress. Adv Appl Microbiol.

[54] Chen L, Luo SL, Xiao X, Guo HJ, Chen JL, Wan Y, Li B, Xu TY, Xi Q, Rao C, Liu CB, Zeng GM (2010). Application of plant growth-promoting endophytes (PGPE) isolated from *Solanum nigrum* L. for phytoextraction of Cd-polluted soils. Appl Soil Ecol..

[55] Mayak S, Tirosh T, Glick BR (2004). Plant growth-promoting bacteria that confer resistance to water stress in tomatoes and peppers. Plant Sci.

[56] Azcon R, El-Atrash F (1997). Influence of arbuscular mycorrhizae and phosphorus fertilization on growth, nodulation and N_2_ fixation (15 N) in Medicago sativa at four salinity levels. Biol Fert Soils..

[57] Augé RM, Toler HD, Saxton AM (2014). Arbuscular mycorrhizal symbiosis and osmotic adjustment in response to NaCl stress-a meta-analysis. Front Plant Sci..

[58] Roychoudhury A, Basu S, Sengupta DN (2010). Amelioration of salinity stress by exogenously applied spermidine or spermine in three varieties of indica rice differing in their level of salt tolerance. J Plant Physiol.

[59] Founoune H, Duponnois R, Baaaa AM, El-Bouami F (2002). Influence of the dual arbuscular endomycorrhizal/ectomycorrhizal symbiosis on the growth of *Acacia holosericea* (A Cunn Ex G Don) in glasshouse conditions. Ann For Sci..

[60] Ojala JC, Jarrel WM, Menge JA, Johnson ELV (1983). Influence of mycorrhizal fungi on the mineral nutrition and yield of onion in saline soil. Agron J..

[61] Plaut Z, Grieve CM (1988). photosynthesis of salt stressed maize as influenced by Ca:Na ratios in the nutrient solution. Plant Soil.

[62] Sirivastava TP, Gupta SC, Lal P, Muralia N, Kumar N (1998). Effect of salt stress on physiological and biochemical parameters of wheat. Ann Arid Zone.

[63] Abdel-Fattah GM (2001). Measurement of the viability of arbuscular-mycorrhizal fungi using three different stains; relation to growth and metabolic activities of soybean plants. Microbiol Res.

[64] Kaul S, Sharma SS, Mehta IK (2008). Free radical scavenging potential of l-proline evidence from in vitro assays. Amino Acids.

[65] Goudarzi M, Pakniyat H (2009). Salinity causes increase in proline and protein contents and peroxidase activity in wheat cultivars. J Appl Sci..

[66] Abdel-Ghani AH (2009). Response of wheat varieties from semi-arid regions of Jordan to salt stress. J Agron Crop Sci.

[67] Aldesuquy HS, Ibrahim AH (2001). Interactive effect of seawater and growth bioregulators on water relations, abscisic acid concentration and yield of wheat plants. J Agron Crop Sci.

[68] Torres CB, Bingham FT (1973). Salt tolerance of Mexican wheat. I. Effect of NO_3_ and NaCl on mineral nutrition, growth and grain production of wheat. Soil Sci.

[69] Glick BR, Cheng Z, Czarny C, Duan J (2007). Promotion of plant growth by ACC deaminase-containing soil bacteria. Eur J Plant Pathol.

[70] Daei G, Ardekani MR, Rejali F, Teimuri S, Miransari M (2009). Alleviation of salinity stress on wheat yield, yield components, and nutrient uptake using arbuscular mycorrhizal fungi under field conditions. J Plant Soil..

[71] Abdel-Fattah GM, Asrar ABA (2012). Arbuscular mycorrhizal fungal application to improve growth and tolerance of wheat (*Triticum aestivum* L.) plants grown in saline soil. Acta Physiol Plant..

[72] Gyaneshwar P, Kumar GN, Parekh LJ, Poole PS (2002). Role of soil microorganisms in improving P nutrition of plants. Plant Soil.

[73] Reinhold-Hurek B, Hurek T (1998). Life in grasses: diazotrophic endophytes. Trends Microbiol.

[74] Sheng XF, Xia JJ, Jiang CY, He LY, Qian M (2008). Characterization of heavy metal-resistant endophytic bacteria from rape (*Brassica napus*) roots and their potential in promoting the growth and lead accumulation of rape. Eviron Pollut..

[75] Zhang X, Li E, Xiong XL, Shen DL, Feng YL (2010). Colonization of endophyte Pantoea agglomerans YS19 on host rice, with formation of multicellular symplasmata. World J Microbiol Biotech..

[76] Shankar M, Ponraj P, Ilakkiam D, Gunasekaran P (2011). Root colonization of a rice growth promoting strain of *Enterobacter cloacae*. J Basic Microbiol.

[77] Sturz AV, Christie BR, Matheson BG (1998). Associations of bacterial endophyte populations from red clover and potato crops with potential foe beneficial allelopathy. Can J Microbiol.

[78] Surette MA, Sturz AV, Rajasekaran R, Lada RR, Nowak J (2003). Bacterial endophytes in processing carrots (*Daucus carota* L. var. sativus): their localization, population density, biodiversity and their effects on plant growth. Plant Soil.

[79] Wu G, Lewis J, Hoffmann C, Chen YY, Knight R, Bittinger K, Hwang J, Chen J, Berkowsky R, Nessel L, Li H, Bushman F (2010). Sampling and pyrosequencing methods for characterizing bacterial communities in the human gut using 16S sequence tags. BMC Microbiol.

[80] Nowell JA, Parules JB (1980). Preparation of experimental tissue for scanning electron microscopy.

[81] Abdel-Malek Y, Ishac YZ (1968). Evaluation of methods used in counting Azotobacter. J Appl Bact..

[82] Jensen HL (1942). Nitrogen fixation in leguminous plants II. Is symbiotic nitrogen fixation influenced by Azotobacter. Pro Line Soc NSW..

[83] Cappuccino JC, Sherman N (1992). Microbiology; a laboratory manual.

[84] Penrose DM, Glick BR (2003). Methods for isolation and characterizating ACC deaminase-containing plant growth promotiong rhizobacteria. Physiol Plant.

[85] Sheng XF, He LY, Wang QY, Ye HS, Jiang CY (2008). Effects of inoculation of biosurfactant-producing *Bacillus* sp. J119 on plant growth and cadmium uptake in a cadmium-amended soil. J Hazard Mater.

[86] Gordon SA, Weber RP (1951). Colorimetric estimation of indoleacetic acid. Plant Physiol.

[87] Schwyn R, Neilands JB (1987). Universal chemical assay for detection and estimation of siderophores. Anal Biochem.

[88] Payne SM, Clark VL, Bovil PM (1994). Detection, isolation, characterization of siderophores. Methods in Enzymology.

[89] Nautiyal CS (1999). An efficient microbiological growth medium for screening phosphate solubilizing microorganisms. FEMS Microbiol Lett.

