# Supplementary material for: The endophytic bacteria isolated from elephant grass (Pennisetum purpureum Schumach) promote plant growth and enhance salt tolerance of Hybrid Pennisetum
Source: Biotechnol Biofuels. 2016 Sep 2;9(1):190. doi: 10.1186/s13068-016-0592-0 (PMC5010695; doi:10.1186/s13068-016-0592-0)
Supplement: Supplementary file 1 — 10.1186/s13068-016-0592-0 The densities of the four endophytic bacterial strains corresponding to different OD600 values 0.3–2.0. [file 13068_2016_592_MOESM1_ESM.docx]

**Additional file 1**

**Table S1.** The densities of the four endophytic bacterial strains corresponding to different OD_600_ values 0.3-2.0.

|  | Endophytic bacterial strain | OD_600_ | CFU mL^-1^ |
| --- | --- | --- | --- |
|  | *Sphingomonas* sp. pp01 | 0.3 | ~ 9.06×10^7^ |
|  |  | 0.7 | ~ 1.85×10^8^ |
|  |  | 1 | ~ 2.32×10^8^ |
|  |  | 1.5 | ~ 2.63×10^8^ |
|  |  | 2 | ~ 2.97×10^8^ |
| *Bacillus* sp. pp02 | | 0.3 | ~ 1.38×10^5^ |
|  |  | 0.7 | ~ 1.92×10^5^ |
|  |  | 1 | ~ 1.21×10^6^ |
|  |  | 1.5 | ~ 5.44×10^6^ |
|  |  | 2 | ~ 2.74×10^7^ |
|  | *Pantoea* sp. pp04 | 0.3 | ~ 2.62×10^7^ |
|  |  | 0.7 | ~ 1.66×10^8^ |
|  |  | 1 | ~ 2.03×10^8^ |
|  |  | 1.5 | ~ 2.28×10^8^ |
|  |  | 2 | ~ 3.01×10^8^ |
|  | *Enterobacter* sp. pp06 | 0.3 | ~ 8.53×10^7^ |
|  |  | 0.7 | ~ 1.92×10^8^ |
|  |  | 1 | ~ 2.12×10^8^ |
|  |  | 1.5 | ~ 2.96×10^8^ |
|  |  | 2 | ~3.38×10^8^ |
